# Dysfunction in Fatty Acid Amide Hydrolase Is Associated with Depressive-Like Behavior in Wistar Kyoto Rats

**DOI:** 10.1371/journal.pone.0036743

**Published:** 2012-05-14

**Authors:** K. Yaragudri Vinod, Shan Xie, Delphine Psychoyos, Basalingappa L. Hungund, Thomas B. Cooper, Shanaz M. Tejani-Butt

**Affiliations:** 1 Division of Analytical Psychopharmacology, Nathan Kline Institute for Psychiatric Research, Orangeburg, New York, United States of America; 2 Department of Child and Adolescent Psychiatry, New York University School of Medicine, New York, New York, United States of America; 3 Center for Environmental and Genetic Medicine, Texas A&M Health Science Center, Houston, Texas, United States of America; 4 Division of Molecular Imaging and Neuropathology, New York State Psychiatric Institute, New York, New York, United States of America; 5 Department of Psychiatry, College of Physicians and Surgeons, Columbia University, New York, New York, United States of America; 6 Department of Pharmaceutical Sciences, University of the Sciences in Philadelphia, Philadelphia, Pennsylvania, United States of America; Sapienza University of Rome, Italy

## Abstract

**Background:**

While the etiology of depression is not clearly understood at the present time, this mental disorder is thought be a complex and multifactorial trait with important genetic and environmental contributing factors.

**Methodology/Principal Findings:**

The role of the endocannabinoid (eCB) system in depressive behavior was examined in Wistar Kyoto (WKY) rat strain, a genetic model of depression. Our findings revealed selective abnormalities in the eCB system in the brains of WKY rats compared to Wistar (WIS) rats. Immunoblot analysis indicated significantly higher levels of fatty acid amide hydrolase (FAAH) in frontal cortex and hippocampus of WKY rats with no alteration in the level of N-arachidonyl phosphatidyl ethanolamine specific phospholipase-D (NAPE-PLD). Significantly higher levels of CB1 receptor-mediated G-protein coupling and lower levels of anandamide (AEA) were found in frontal cortex and hippocampus of WKY rats. While the levels of brain derived neurotropic factor (BDNF) were significantly lower in frontal cortex and hippocampus of WKY rats compared to WIS rats, pharmacological inhibition of FAAH elevated BDNF levels in WKY rats. Inhibition of FAAH enzyme also significantly increased sucrose consumption and decreased immobility in the forced swim test in WKY rats.

**Conclusions/Significance:**

These findings suggest a critical role for the eCB system and BDNF in the genetic predisposition to depressive-like behavior in WKY rats and point to the potential therapeutic utility of eCB enhancing agents in depressive disorder.

## Introduction

Major depressive disorder (MDD) is characterized by a significant impairment in mood and motivation [1], and exhibits a chronic, relapsing course and is associated with high morbidity and mortality worldwide. Depression is the leading cause of disability and the 4th leading contributor to the global burden of disease in 2000 [2]. In the United States alone, more than 30,000 people commit suicide each year; majority of which are associated with depression [2], [3]. Although the etiology of this disorder is not clearly understood, clinical observations suggest a significant role for the monoamine neurotransmitter systems [4]. However, currently used antidepressants, which alter the monoamine systems, appear to be therapeutically inadequate in many patients. Thus, further studies are needed to understand the pathophysiological basis of depression and for developing more effective therapeutic agents.

Recent studies have implicated the eCB system in neuropsychiatric disorders including depression and suicide [5]. A potential role for the brain eCB system in the pathophysiology of MDD was initially demonstrated in a post-mortem study that showed upregulation of CB1 receptor in dorsolateral prefrontal cortex (DLPFC) of depressed suicide victims [6]. Since then, a number of studies have examined the role of the eCB system in the neurobiology of depression; however, the findings have been contradictory in that antidepressant-like properties have been reported for both CB1 receptor agonist as well as the antagonist [7]–[14]. Given that depressive disorder is a complex and multifactorial trait with important genetic and environmental contributing factors, several genetic animal models have been developed in order to identify factors that underlie predisposition to depression and to develop pharmacotherapy [15], [16]. Previous studies have established Wistar Kyoto (WKY) rat as an important animal model of depressive disorder [15], [17]–[21]. In the present study, we investigated whether dysfunction in the brain eCB system is associated with depressive-like behavior in WKY rat. To further understand the molecular mechanisms downstream of the eCB system, the effect of FAAH inhibition on BDNF was also investigated as it has been shown to be critically involved in the etiology of major depression and in antidepressant effects [22].

## Materials and Methods

### Animals

WKY and WIS rats (10–12 week old male rats) used for this study were procured from Charles River laboratories and bred at the Animal Facility of the Nathan Kline Institute (NKI). Rats were housed at 23±1°C for 12 h light/dark cycle in a group of two rats and habituated to environment and handling for a week prior to the experiments. Animal care and handling procedures were done in accordance with the Institutional and NIH guidelines. The animal care protocol was approved by the Institutional Animal Care and Use Committee of the NKI (# AP2009-297). For basal comparison, WKY rats and the control WIS rats were euthanized under anesthesia (chlorate hydrate 400 mg/kg, i.p.) and brain regions (frontal cortex and hippocampus) were dissected on ice. Brain regions were used for the analysis of AEA, FAAH, CB1 receptor, CB1 receptor-mediated G-protein activation and BDNF. The effects of pharmacological inhibition of FAAH (URB597, 0.3 mg/kg body wt, i.p.) for 7 days on depressive-like phenotype, AEA, CB1 receptor-mediated G-protein activation and BDNF levels were also examined in WKY rats compared to vehicle treated WKY rats.

**Figure 1:**
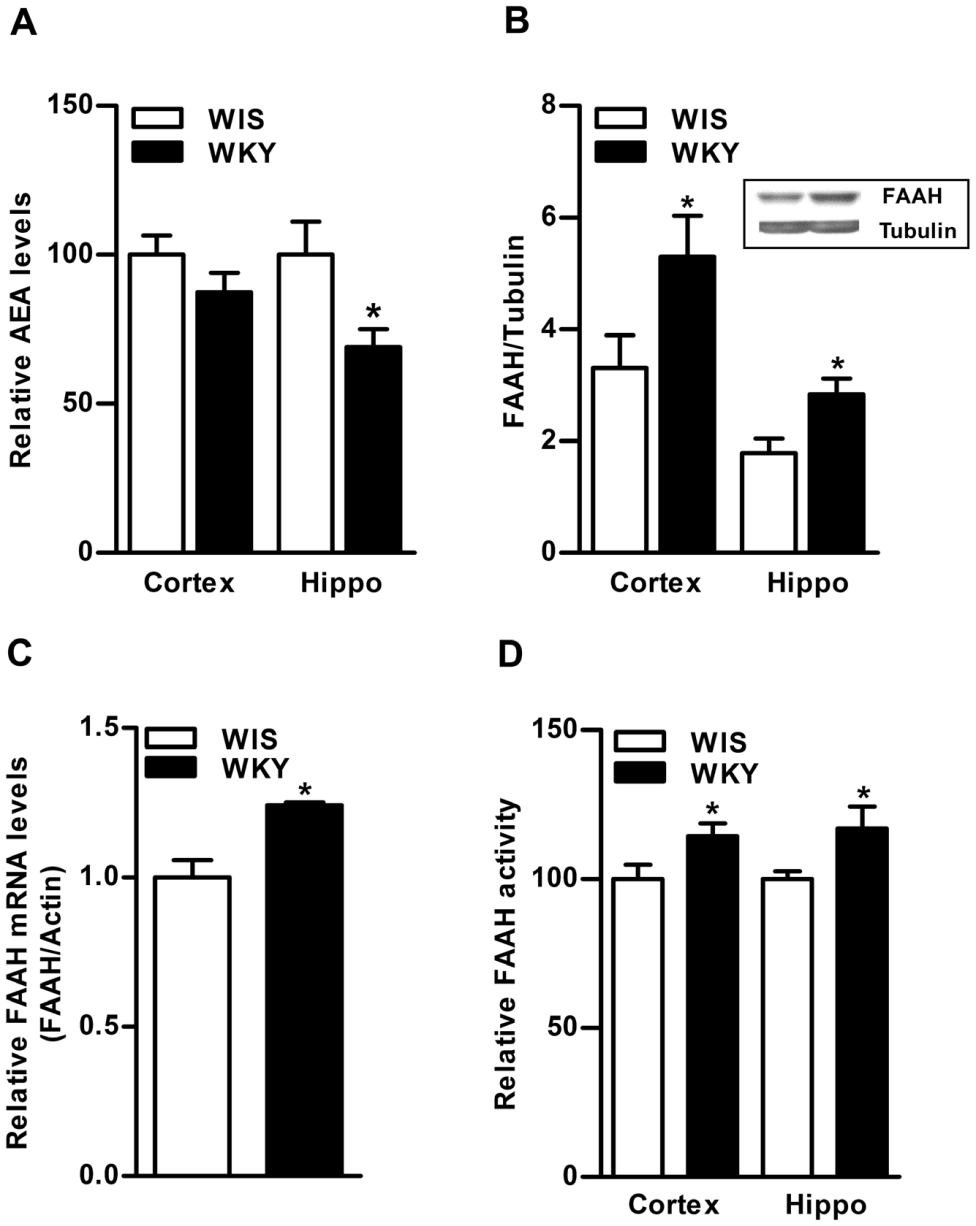
Basal differences in AEA and FAAH levels in the brain of WKY rats. The level of eCB, AEA was found to be significantly lower in hippocampus of WKY rats compared to WIS rats (31%, p<0.01; A). Conversely, the level of FAAH enzyme was significantly higher in frontal cortex (40%, p<0.05) and hippocampus (40%, p<0.05; B) of WKY rats. A representative immunoblot for hippocampus is provided in the upper panel (B). The qPCR analysis also indicated higher levels of mFAAH in hippocampus of WKY rats (24%, p<0.05; C). The qPCR data on FAAH, normalized to β-Actin (internal standard) is presented as the fold change relative to the control value of 1.0. The FAAH activity was slightly higher in frontal cortex (15%, p<0.05) and hippocampus (17%, p<0.05) of WKY rats compared to WIS rats (D). Hippo; Hippocampus.

**Figure 2:**
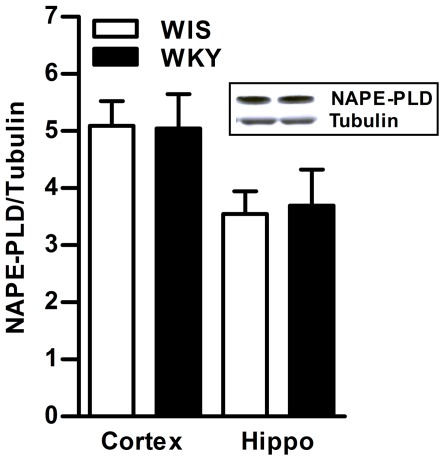
Basal levels of NAPE-PLD in the brain of WKY rats. There were no significant differences in the levels of NAPE-PLD enzyme in frontal cortex and hippocampus of WKY rats compared to WIS rats (A). A representative immunoblot for hippocampus is provided in the upper panel (B). Hippo; Hippocampus.

**Figure 3:**
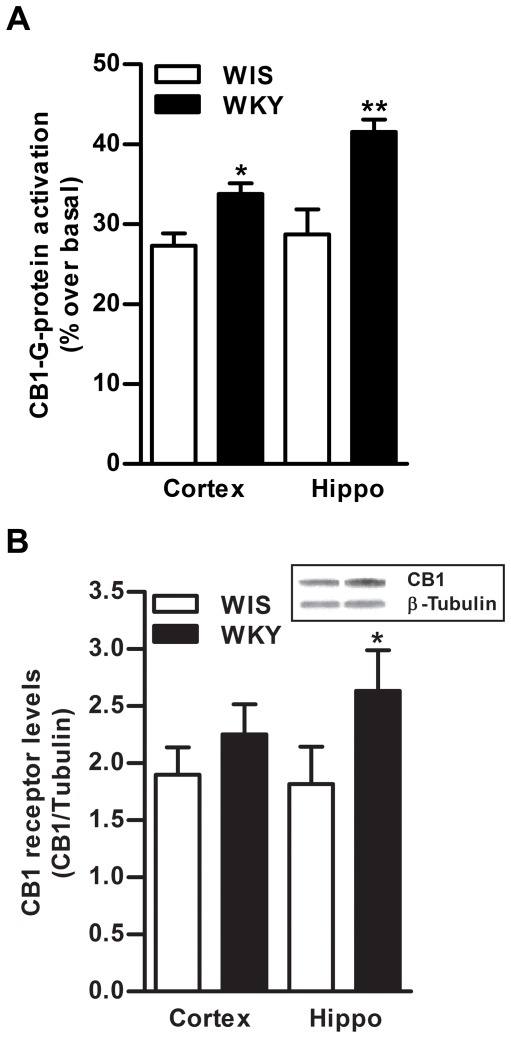
Basal differences in CB1 receptor in the brain of WKY rats. The CB1 receptor agonist-stimulated [^35^S]GTPγS binding was significantly higher in frontal cortex (24%, p<0.05) and hippocampus (44%, p<0.01) of WKY rats compared to WIS rats (A). Data is presented as percentage of stimulation over basal binding. Western blot analysis revealed significantly higher levels of CB1 receptors in hippocampus (45%, p<0.05), while they were found to be slightly higher in frontal cortex of WKY rats (18%, B). Hippo; Hippocampus.

### AEA Assay

Levels of eCB, AEA, were determined using liquid chromatography mass spectroscopy (LC-MS) following the isotopic dilution procedure described previously [23]. Briefly, tissue was homogenized in 4 ml of chloroform-methanol-tris buffer (2∶1∶1, pH 7.4) containing 0.25 mM PMSF, 0.2% BHT, 50 ng of AEA-d_8_. The homogenate was centrifuged at 1,000 g and the organic layer was taken to dryness with nitrogen. The residue was dissolved in ethyl acetate (0.3 ml) and centrifuged. The supernatant was dried and the residue was redissolved in alcohol (30 µl) and transferred to a vial for the measurement of AEA by LC-MS. The standard curve was fitted with a quadratic equation with the curve encompassing a range of 1–50 ng and was processed similarly with quality controls with each batch of samples.

**Figure 4:**
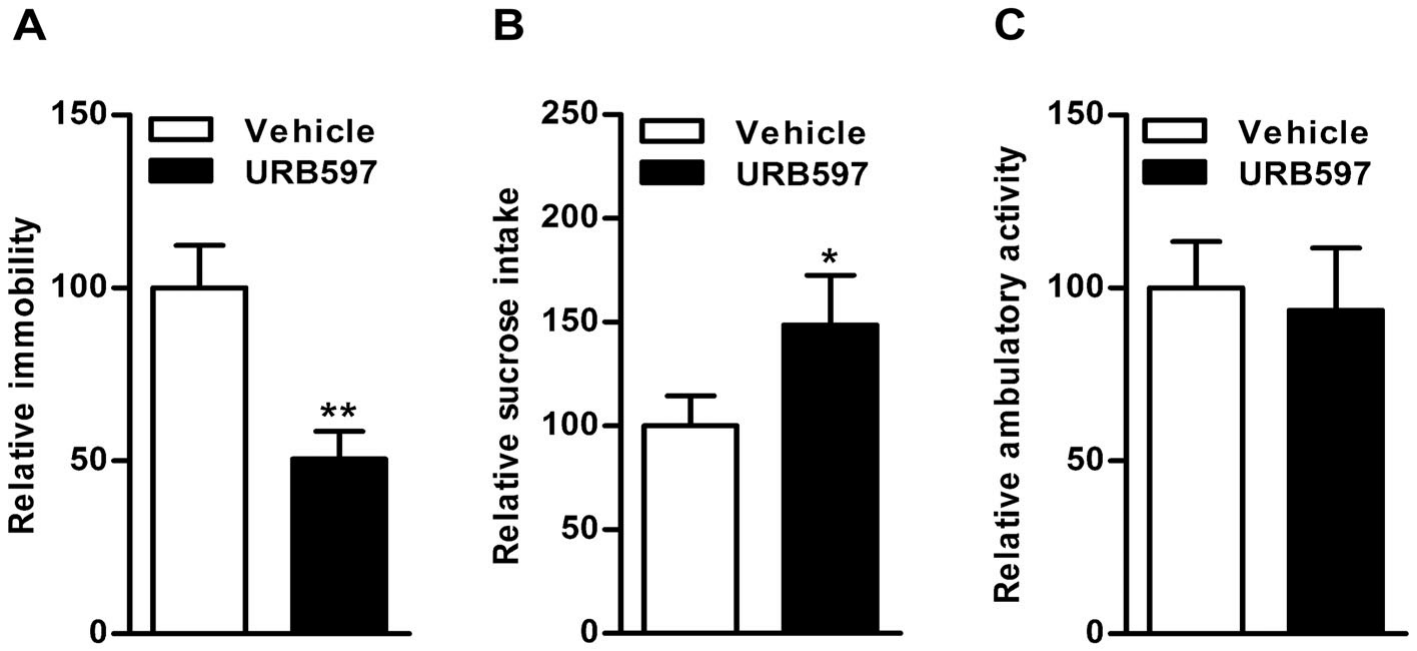
Effect of FAAH inhibition on depressive-like behavior in WKY rats. Treatment with URB597 (0.3 mg/kg, i.p. for 7 days) elicited a significant decrease in total time spent in immobility (50%, p<0.01; A) and a marked increase in sucrose intake (48%, p<0.05; B) without any effect on the spontaneous locomotor activity in the open field (C) in WKY rats compared to vehicle treated WKY rats.

### Immunoblot Analysis

An aliquot of tissue homogenate (30 µg protein) was electrophoresed using 12% polyacrylamide gel and transferred to nitrocellulose membrane. Membrane was treated with blocking buffer (TTBS, [10 mM Tris, 0.9% NaCl; 1% Tween 20 containing 5% milk powder] of pH 7.4) for 1 hr at room temperature. Membrane was then incubated with antibodies for FAAH, NAPE-PLD and CB1 receptor, (Abnova, Taipei City, Taiwan) overnight at 4°C. The blot was washed with TTBS and then incubated with HRP conjugated anti-IgG for 1 hr at room temperature. After washing the blot with TTBS, the immunoreactive band was visualized using ECL reagent (GE Health Care, Piscataway, NJ). The blot was reprobed with tubulin antibody to ensure equal protein loading.

**Figure 5:**
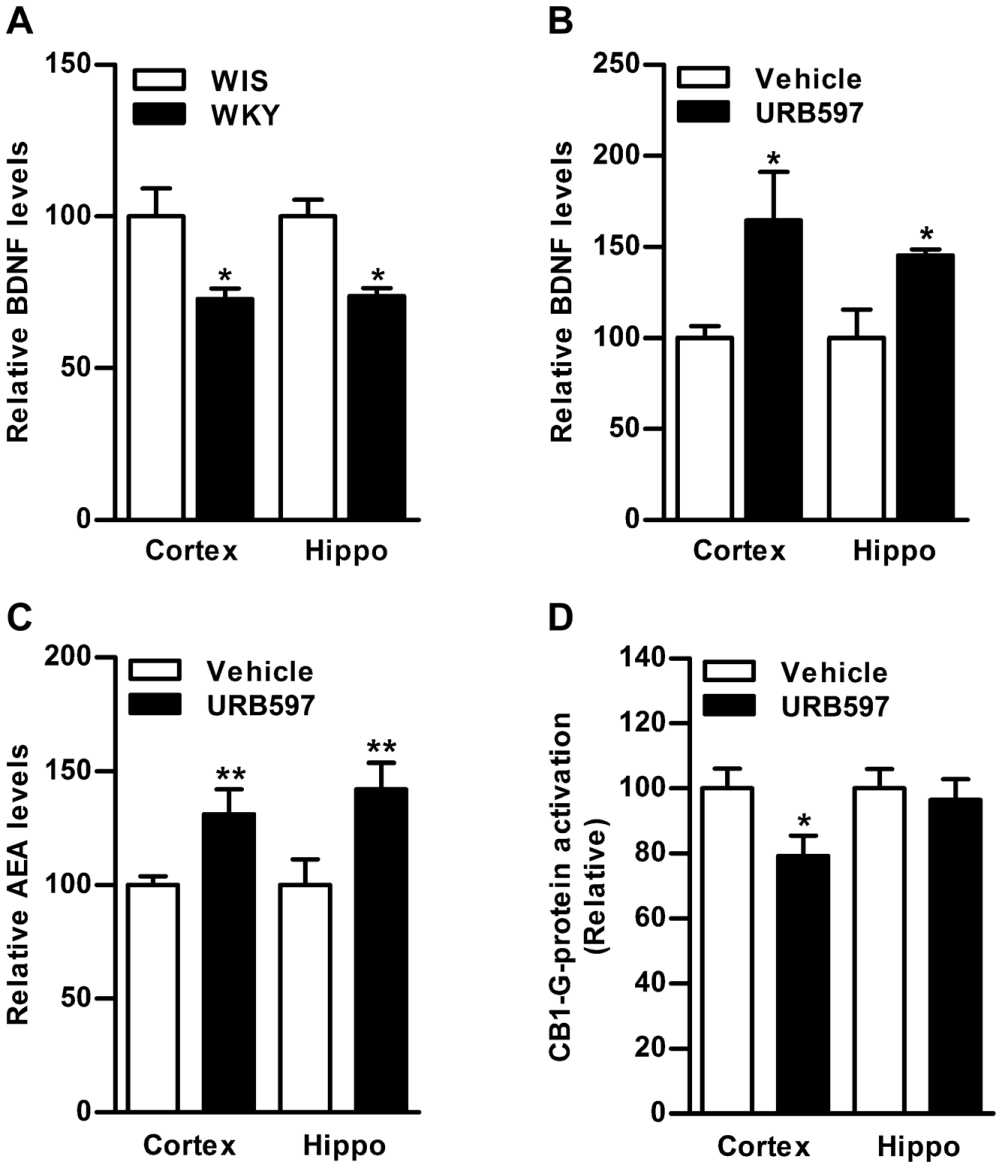
Effect of FAAH inhibition on BDNF, AEA and CB1 function in the brain of WKY rats. Basal BDNF levels were found to be significantly lower in frontal cortex (27%) and hippocampus (26%) of WKY compared to WIS rats (p<0.05; A). Subchronic treatment with URB597 (0.3 mg/kg, i.p. for 7 days) significantly elevated BDNF levels in frontal cortex (64%) and hippocampus (45%) of WKY rats compared to vehicle treated WKY rats (p<0.05; B). Inhibition of FAAH was accompanied by significant increase in AEA levels in frontal cortex (31%, p<0.01; C) and hippocampus (42%, p<0.001; C), and a subsequent decrease in CB1 receptor-mediated G-protein activation in frontal cortex of WKY rats (21%, p<0.05; D). Hippo; Hippocampus.

### Real-time Quantitative PCR (qPCR) Studies with FAAH

Total RNA was extracted using Ambion AqueousRNA-4PCR kit (Life Technologies, Carlsbad, CA). RNA quality and concentration were measured using a ND-1000 instrument (Thermo Fisher Scientific, USA) and a 1% agarose gel. For cDNA synthesis, 1 µg RNA from each sample was reverse transcribed to cDNA using High Capacity RNA-to-cDNA Kit (Life Technologies, Carlsbad, CA). qPCR was performed using Gene Expression Assays FAAH (Rn00577086_m1) and ActB (Rn00667869_m1) on a ABI Prism 7900 HT instrument (Life Technologies, Carlsbad, CA). Reactions were set on 384-well plates (BioRad, Hercules, CA) in a volume of 20 µl containing 100 ng of cDNA template, 1 µl Gene Expression Assay and 10 µl TaqMan Gene Expression Master Mix (Life Technologies, Carlsbad, CA). A no-template control (NTC) was performed for each primer set used. The thermal profile was as follows: 2 minutes at 95°C, followed by 45 cycles of amplification where each cycle comprised of 12 seconds at 95°C and 60 seconds at 60°C. Each sample was assayed in triplicate. The qPCR data was analyzed using SDS2.4 software (Life Technologies, Carlsbad, CA).

### Measurement of FAAH Activity

The FAAH activity was measured as described previously [7], [24]. Briefly, tissue homogenate (25–50 µg of total protein) was incubated with 30 µM AEA (ethanolamine1-^3^H) (10–20 Ci/mmol) in a solution containing 0.1 M Tris-HCl (pH 8.0), 0.1% BSA, for 30 min at 37°C. After incubation, samples were extracted by organic solvent (chloroform and methanol; 1∶1) and subjected to liquid scintillation counting.

### Agonist-stimulated [^35^S]GTPγS Binding Assay

The [^35^S]GTPγS binding assay was performed in crude synaptic membrane isolated from frontal cortex and hippocampus as described previously [24]. Briefly, all ligands were diluted in assay buffer (50 mM Tris-HCl, 3 mM MgCl_2_, 100 mM NaCl, 1 mM EDTA) containing 0.1 mg/ml fatty acid-free BSA. The assay mixture was incubated in silicone-treated test tubes for 1 hr at 30°C. Reaction was terminated by adding 2 ml of ice-cold Tris-HCl buffer. Membranes were rapidly filtered through GF/B filters using a Brandel 48-position cell harvester and were washed with ice-cold wash buffer (50 mM Tris-HCl). The filters were transferred to scintillation vials containing 5 ml of scintillation cocktail and the radioactivity was measured using a liquid scintillation counter at an efficiency of 95% for ^35^S. Non-specific binding was determined by addition of 100 µM unlabeled GTPγS. The CB1 antagonist (SR141716A) was used to study the specificity of CB1 agonist [CP-55,940; 1 µM] stimulated [^35^S]GTPγS binding.

### Forced-swim Test, Sucrose Intake and Spontaneous Motor Activity

Antidepressant-like property of URB597 was evaluated using the forced-swim test (FST) as it is a sensitive and reliable method with high predictive validity [7], [25]. The dose and duration of treatment were selected based on the literature [7]. WKY rats were treated with FAAH inhibitor, URB597 (0.3 mg/kg body wt, i.p.) once daily in the morning (10 AM) for 7 days. The control WKY rats received vehicle (saline containing 1% DMSO and 1% Tween 20). After 3 hr following the administration of last dose of URB597, rats were tested for FST and sucrose consumption [7]. During the 30 min swim test, the rat behavior was videotaped. The main behaviors, immobility (no or minimum movement), swimming and climbing were assessed. A potential drug-induced change in the spontaneous locomotor activity in an open field (Columbus Instruments, Columbus, OH) was also measured for 30 min. For the sucrose consumption test, rats were housed in individual cages and offered access to preweighed bottles containing tap water and 1% sucrose 3 hr after the last injections of URB597. The amount of water and sucrose consumption was measured for 2 hr.

### BDNF Levels

Basal levels of BDNF were measured using Sandwich Elisa Kit (Millipore, Temecula, CA) in frontal cortex and hippocampus of WKY and WIS rats. The effect of URB597 (0.3 mg/kg body wt, i.p.) treatment for 7 days on BDNF levels were also measured in WKY rats compared to vehicle treated WKY rats after 3 hr following the last dose of URB597.

### Statistical Analysis

The statistical analyses were performed using independent student “t” test (GraphPad Software, San Diego, CA). All the statistical analyses were run on the raw data. The data on innate differences in the eCB system and BDNF between the groups (WKY and WIS rats) were analyzed using unpaired “t” test. Paired “t” tests were applied for the analysis of the data on the effect of pharmacological treatment on depressive-like behavior, spontaneous activity, sucrose consumption and BDNF levels in WKY rats compared to vehicle treated WKY rats. The qPCR data on FAAH, normalized to β-Actin (endogenous reference) was given by 2^−ΔΔCt^. Statistical differences were considered to be significant at p<0.05. The values (mean±SEM) are presented as percentage over the control groups or otherwise stated.

## Results

### Basal Differences in the Components of eCB System in the Brains of WIS and WKY Rats

Basal levels of eCB, AEA were found to be significantly lower in hippocampus of WKY rats compared to WIS rats (31%, p<0.01, n = 5−6; Fig. 1A). Basal level of FAAH enzyme was significantly higher in frontal cortex (40%, p<0.05) and hippocampus (40%, p<0.05) of WKY rats compared to WIS rats (n = 6 in each group; Fig. 1B). A representative immunoblot is provided in the upper panel (Fig. 1B). The qPCR analysis also confirmed higher levels of mFAAH in hippocampus of WKY rats than WIS rats (24%, p<0.05; Fig. 1C; n = 4). A subtle but statistically significant higher activity of FAAH enzyme was observed in frontal cortex (15%, p<0.05) and hippocampus (17%, p<0.05; n = 6−8; Fig. 1D) of WKY rats than WIS rats. There were no significant differences in the levels of NAPE-PLD enzyme in frontal cortex and hippocampus of WKY compared to WIS rats (Fig. 2). A representative immunoblot is provided in the upper panel (Fig. 2). The CB1 receptor-stimulated [^35^S]GTPγS binding was significantly higher in frontal cortex (24%, p<0.05) and hippocampus (44%, p<0.01) of WKY rats compared to WIS rats (n = 6−8; Fig. 3A). Western blot analysis revealed significantly higher levels of CB1 receptors in hippocampus (45%, p<0.05), however, they were slightly higher in frontal cortex of WKY rats (18%, n = 6−8; Fig. 3B).

### Effect of FAAH Inhibition on FST and Sucrose Intake

Pharmacological inhibition of FAAH with URB597 (0.3 mg/kg, i.p. for 7 days) elicited a significant decrease in total time spent in immobility (50%, p<0.01; Fig. 4A) and increased sucrose intake (48%, p<0.05; Fig. 4B) without affecting the spontaneous locomotor activity in the open field in WKY rats compared to vehicle treated WKY rats (n = 5−8; Fig. 4C).

### Effect of FAAH Inhibition on AEA, CB1 Receptor Function and BDNF

Basal levels of BDNF were found to be significantly lower in frontal cortex (27%) and hippocampus (26%) of WKY rats compared to WIS rats (p<0.05; n = 4−6; Fig. 5A). Subchronic URB597 treatment (0.3 mg/kg, i.p. for 7 days) markedly elevated BDNF levels in frontal cortex (64%) and hippocampus (45%) of WKY rats compared to vehicle treated WKY rats (p<0.05; n = 4−6; Fig. 5B). This treatment was accompanied by significant increase in AEA levels in frontal cortex (31%, p<0.01; Fig. 5C) and hippocampus (42%, p<0.001; Fig. 5C), and decrease in CB1 receptor-mediated G-protein activation (21%, p<0.05; Fig. 5D) in frontal cortex of WKY rats compared to vehicle treated WKY rats (n = 4−6).

## Discussion

Previous behavioral and biochemical studies have established the WKY rat as an important genetic animal model of depressive behavior [15]–[21]. To our knowledge, the present study is the first to explore the role of the eCB system in this model. The findings revealed a higher CB1 receptor-mediated G-protein activation in frontal cortex and hippocampus of WKY rats compared to the control strain, WIS rats. This is in agreement with our previous study that reported higher levels of CB1 receptor-mediated G-protein coupling in DLPFC of depressed patients [6]. While CB1 receptors were not found to be significantly higher in frontal cortex of WKY rats, the potential changes in G-protein levels and brain regions of interest (DLPFC versus frontal cortex) might be contributing factors for this discrepancy. Alterations in the metabolic enzymes of eCBs due to stress or any other insults could alter eCB tone leading to adaptive changes in CB1 receptor signaling. In animal studies, exposure to stress is shown to reduce eCB levels and upregulate mRNA of CB1 receptor [26], [27]. Notably, we found significantly lower AEA levels in hippocampus of WKY rats compared to WIS rats. Therefore, sensitization of CB1 receptor might be a compensatory adaptation in response to diminished eCB tone. The reduction in AEA levels appears to be mainly due to higher activity of FAAH enzyme in WKY rats compared to WIS rats. To understand a relevance of upregulation of CB1 receptors/G-protein activation to depressive behavior, we examined CB1 receptor-mediated G-protein coupling following drug treatment. Subchronic FAAH inhibition led to a subtle but statistically significant reduction in CB1 receptor-mediated G-protein activation in frontal cortex of WKY rats. This desensitization is likely due to a neuroadaptation to persistent elevation of AEA and activation of CB1 receptors. It remains to be determined if chronic (or higher dose) of URB597 treatment is required to attenuate hippocampal CB1 receptors in WKY rats. The observed effects are likely mediated through CB1 receptors, since several previous studies have demonstrated that URB597 treatment elevates AEA and exerts its effect through CB1 receptors in other rodent models [7], [28]–[32].

We further investigated whether elevation of AEA through inhibition of FAAH activity has an antidepressant-like property in WKY rats. The rationale for selecting FAAH inhibitor over CB1 receptor agonist is that (1) FAAH level and activity was found to be higher in brain of WKY rats in the present study and (2) direct modifications of CB1 receptor signaling using CB1 receptor agonists have shown to exert variable side effects [27]. Our results demonstrate that subchronic inhibition of FAAH with URB597 elicits a significant decrease in total time spent in immobility without any effect on spontaneous locomotor activity in the open field in WKY rats. Inhibition of FAAH by URB597 is reported to enhance the accumulation of AEA after 2 hrs of treatment and produces an antidepressant-like effect in a rat model of subchronic mild stress [7], [10]. The cannabinoids have been shown to elicit antidepressant-like behavior, probably through the activation of serotonergic neurons in medial prefrontal cortex [9]. It remains to be seen if antidepressant-like property of URB597 in WKY rats is linked to an increase in brain serotonergic system. Furthermore, polymorphism in FAAH gene is shown to be associated with bipolar disorder and major depression [33]. However, it is yet to be determined if such a polymorphism is related to an increase in the expression and/or activity of FAAH.

Our study further demonstrates that pharmacological inhibition of FAAH enzyme significantly increases the sucrose consumption in WKY rats. Increase in sucrose intake following FAAH inhibition appears to be related to a decrease in hedonic response leading to increased sensitivity to reward. It is interesting to note that enhancement of the CB1 receptor-mediated signaling in hippocampus elicits an antidepressant-like effect in rodents [34], [35], suggesting an association of reduction in the eCB signaling with stress and depressive behavior. Conversely, there are a number of studies where both agonist and antagonist of CB1 receptors have been shown to act as antidepressants [5]–[14], [27]. These discrepancies may be due to differences in animal species, strains used, behavioral paradigms (e.g. FST *vs* CMS or learned despair etc), drugs or dosages used in various studies. In addition, interspecies or interstrain differences in brain regional distribution of the eCB system may also influence the behavioral outcome.

Recent imaging studies in humans have provided information about the neuroanatomical correlates of mood disorders. The biochemical and morphological changes in prefrontal cortex and hippocampus are reported in patients with mood disorders. The hippocampus is an important brain region of the limbic stress pathway and a major feedback site for glucocorticoids in response to stress [36]–[42]. Stress has also been shown to adversely affect cortical and hippocampal function by deregulating expression of neurotrophic factors that promote neuronal plasticity. For instance, an etiological link between the development of depression and BDNF has been suggested [22]. Exposure to stress is shown to decrease the expression of BDNF while antidepressant treatment and electroconvulsive therapy increase the expression of BDNF [43]–[52]. Our findings have revealed for the first time the existence of lower levels of BDNF in frontal cortex and hippocampus of WKY rats compared to WIS rats. Furthermore, BDNF appears to be under the regulation of AEA-mediated signaling since FAAH inhibition elevated its level in WKY rats. Consistent with these observations, previous studies have shown that CB1 knockout mice exhibit an augmented response to stress (increased despair behavior and corticosterone) with decreased BDNF levels in hippocampus [53]. Notably, local administration of BDNF in hippocampus reversed the increased despair behavior of CB1 knockout mice. The cannabinoids appear to elicit antidepressant-like effects through promotion of hippocampal neurogenesis [54]. It remains to be seen if eCB-mediated BDNF function promotes neuronal plasticity leading to attenuation of depressive-like behavior in WKY rats. Although the role of BDNF in depressive behavior is yet to be clearly understood, a potential role of genetic variation in BDNF and antidepressant treatment outcome in depression has been reported [43], [55].

The cAMP-CREB pathway is a target of monoamines and several other neuromodulatory systems, and may play a pivotal role in neuronal plasticity associated with stress and depression [56], [57]. The CB1 receptor is among the most abundant neuromodulatory GPCRs in brain, and is coupled to adenylyl cyclase and ERK via Gi/o-protein. It is possible that alterations in the CB1 receptor-mediated G-protein activation could change cAMP content, subsequently affecting the activity of PKA-CREB pathway and gene regulation. In addition, modulation of the hypothalamus-pituitary axis by the eCB system and antidepressant-like properties of the cannabinoid drugs further support involvement of this system in the pathophysiology of depression [5]. Taken together, our study demonstrates the selective abnormalities in the eCB system of WKY rats and further suggests the potential therapeutic utility of AEA enhancing agents in the treatment of depressive behavior. Consistent with previous studies that reported a potential contribution of gene variants in CB1 receptor and FAAH enzyme to the susceptibility of depressive behavior [58], [59], the present findings further corroborate a critical role of the eCB system in genetic model of depressive behavior. Future studies investigating other components of the eCB system in additional limbic brain regions such as striatum and amygdala will further our understanding of the pathophysiology of depression. It also remains to be examined whether AEA enhancing agents will provide a beneficial effect in the treatment-resistant depressive disorder.

## References

[1] Diagnostic, Statistical manual of Mental Disorders: DSM-IV (2004). (American Psychiatric Association, Washington, DC).

[2] World Health Organization website.. http://www.who.int/mental_health/management/depression/definition/en/index.html.

[3] Goldsmith SK, Pellmar TC, Kleinman AM, Bunney WE (2002). *Reducing Suicide: A National Imperative*..

[4] Owens MJ (2004). Selectivity of antidepressants: from the monoamine hypothesis of depression to the SSRI revolution and beyond.. J Clin Psychiatry.

[5] Vinod KY, Hungund BL (2006). Role of the endocannabinoid system in depression and suicide.. Trends Pharmacol Sci.

[6] Hungund BL, Vinod KY, Kassir SA, Basavarajappa BS, Yalamanchili R (2004). Upregulation of CB1 receptors and agonist-stimulated [^35^S]GTPγS binding in the prefrontal cortex of depressed suicide victims.. Mol Psychiatry.

[7] Gobbi G, Bambico FR, Mangieri R, Bortolato M, Campolongo P (2005). Antidepressant-like activity and modulation of brain monoaminergic transmission by blockade of anandamide hydrolysis.. Proc Natl Acad Sci USA.

[8] Hill MN, Gorzalka BB (2005). Is there a role for the endocannabinoid system in the etiology and treatment of melancholic depression?. Behav Pharmacol.

[9] Bambico FR, Katz N, Debonnel G, Gobbi G (2007). Cannabinoids elicit antidepressant-like behavior and activate serotonergic neurons through the medial prefrontal cortex.. J Neurosci.

[10] Bortolato M, Mangieri RA, Fu J, Kim JH, Arguello O (2007). Antidepressant-like activity of the fatty acid amide hydrolase inhibitor URB597 in a rat model of chronic mild stress.. Biol Psychiatry.

[11] Shearman LP, Rosko KM, Fleischer R, Wang J, Xu S (2003). Antidepressant-like and anorectic effects of the cannabinoid CB1 receptor inverse agonist AM251 in mice.. Behav Pharmacol.

[12] Griebel G, Stemmelin J, Scatton B (2005). : Effects of the cannabinoid CB1 receptor antagonist rimonabant in models of emotional reactivity in rodents.. Biol Psychiatry.

[13] Tzavara ET, Davis RJ, Perry KW, Li X, Salhoff C (2003). The CB1 receptor antagonist SR141716A selectively increases monoaminergic neurotransmission in the medial prefrontal cortex: implications for therapeutic actions.. Br J Pharmacol.

[14] Witkin JM, Tzavara ET, Davis RJ, Li X, Nomikos GG (2005). A therapeutic role for cannabinoid CB1 receptor antagonists in major depressive disorders.. Trends Pharmacol Sci.

[15] Pare W, Tejani-Butt SM (2003). Chapter: *Depression: Behavior and Brain: Insights from an Animal Model.*.

[16] Samuels BA, Leonardo ED, Gadient R, Williams A, Zhou J (2011). Modeling treatment-resistant depression.. Neuropharmacology.

[17] Tejani-Butt SM, Pare WP, Yang J (1994). Effect of repeated novel stressors on depressive behavior and brain norepinephrine receptor system in Sprague-Dawley and Wistar Kyoto (WKY) rats.. Brain Res.

[18] Pearson KA, Stephen A, Beck SG, Valentino RJ (2006). Identifying genes in monoamine nuclei that may determine stress vulnerability and depressive behavior in Wistar-Kyoto rats.. Neuropsychopharmacol.

[19] Will CC, Aird F, Redei EE (2003). Selectively bred Wistar-Kyoto rats: an animal model of depression and hyper-responsiveness to antidepressants.. Mol Psychiatry.

[20] De La Garza R, Mahoney JJ (2004). A distinct neurochemical profile in WKY rats at baseline and in response toacute stress: implications for animal models of anxiety and depression.. Brain Res.

[21] Malkesman O, Braw Y, Maayan R Weizman A, Overstreet DH (2006). Two different putative genetic animal models of childhood depression.. Biol Psychiatry.

[22] Martinowich K, Manji H, Lu B (2007). New insights into BDNF function in depression and anxiety.. Nat Neurosci.

[23] Vinod KY, Arango V, Xie S, Kassir SA, Mann JJ (2005). Elevated levels of endocannabinoids and CB1 receptor-mediated G-protein signaling in the prefrontal cortex of alcoholic suicide victims.. Biol Psychiatry.

[24] Vinod KY, Kassir SA, Hungund BL, Cooper TB, Mann JJ (2010). Selective alterations of the CB1 receptors and the fatty acid amide hydrolase in the ventral striatum of alcoholics and suicides.. J Psychiatric Res.

[25] Rittenhouse PA, Lopez-Rubalcava C, Stanwood GD, Lucki I (2002). Amplified behavioral and endocrine responses to forced swim stress in the Wistar-Kyoto rat.. Psychoneuroendocrinol.

[26] Hill MN, Kambo JS, Sun JC, Gorzalka BB, Galea LA (2006). Endocannabinoids modulate stress-induced suppression of hippocampal cell proliferation and activation of defensive behaviours.. Eur J Neurosci.

[27] Mangieri RA, Piomelli D (2007). Enhancement of endocannabinoid signaling and the pharmacotherapy of depression.. Pharmacol Res.

[28] Hill MN, Carrier EJ, McLaughlin RJ, Morrish AC, Meier SE (2008). Regional alterations in the endocannabinoid system in an animal model of depression: effects of concurrent antidepressant treatment.. J Neurochem.

[29] Umathe SN, Manna SS, Jain NS (2011). Involvement of endocannabinoids in antidepressant and anti-compulsive effect of fluoxetine in mice.. Behav Brain Res.

[30] Hill MN, McLaughlin RJ, Morrish AC, Viau V, Floresco SB (2009). Suppression of amygdalar endocannabinoid signaling by stress contributes to activation of the hypothalamic-pituitary-adrenal axis.. Neuropsychopharmacol.

[31] Kinsey SG, O’Neal ST, Long JZ, Cravatt BF, Lichtman AH (2011). Inhibition of endocannabinoid catabolic enzymes elicits anxiolytic-like effects in the marble burying assay.. 32Pharmacol Biochem Behav.

[32] Rossi S, De Chiara V, Musella A, Sacchetti L, Cantarella C (2010). Preservation of striatal cannabinoid CB1 receptor function correlates with the antianxiety effects of fatty acid amide hydrolase inhibition.. Mol Pharmacol.

[33] Monteleone P, Bifulco M, Maina G, Tortorella A, Gazzerro P (2010). Investigation of CNR1 and FAAH endocannabinoid gene polymorphisms in bipolar disorder and major depression.. Pharmacol Res.

[34] Carrier EJ, Patel S, Hillard CJ (2005). Endocannabinoids in neuroimmunology and stress.. Curr Drug Targets CNS Neurol Disord.

[35] McLaughlin RJ, Hill MN, Morrish AC, Gorzalka BB (2007). Local enhancement of cannabinoid CB1 receptor signalling in the dorsal hippocampus elicits an antidepressant-like effect.. Behav Pharmacol.

[36] Sheline Gado MH, Kraemer HC (2003). Untreated depression and hippocampal volume loss.. Am J Psychiatry.

[37] Herman JP, Cullinan WE (1997). Neurocircuitry of stress: central control of the hypothalamo-pituitary-adrenocortical axis.. Trends Neurosci.

[38] Sapolsky RM (2000). Glucocorticoids and hippocampal atrophy in neuropsychiatric disorders.. Arch Gen Psychiatry.

[39] McEwen BS (2001). Plasticity of the hippocampus: adaptation to chronic stress and allostatic load.. Ann NY Acad Sci.

[40] Neumeister A, Charney DS, Drevets WC (2005). Depression and the Hippocampus.. Am J Psychiatry.

[41] Santarelli L, Saxe M, Gross C, Surget A, Battaglia F (2003). Requirement of hippocampal neurogenesis for the behavioral effects of antidepressants.. Science.

[42] Malberg JE, Schechter LE (2005). Increasing hippocampal neurogenesis: a novel mechanism for antidepressant drugs.. Curr Pharm Des.

[43] Ribeiro L, Busnello JV, Cantor RM, Whelan F, Whittaker P (2007). : The brain-derived neurotrophic factor rs6265 (Val66Met) polymorphism and depression in Mexican-Americans.. Neuroreport.

[44] Dwivedi Y (2009). “Brain-derived neurotrophic factor: role in depression and suicide”.. Neuropsychiatric Dis Treat.

[45] Haenisch B, Bilkei-Gorzo A, Caron MG, Bonisch H (2009). Knockout of the norepinephrine transporter and pharmacologically diverse antidepressants prevent behavioral and brain neurotrophin alterations in two chronic stress models of depression.. J Neurochem.

[46] Russo-Neustadt AA, Beard RC, Huang YM, Cotman CW (2000). “Physical activity, and antidepressant treatment potentiate the expression of specific brain-derived neurotrophic factor transcripts in the rat hippocampus”.. Neuroscience.

[47] Shimizu E, Hashimoto K, Okamura N, Koike K, Komatsu N (2003). “Alterations of serum levels of brain-derived neurotrophic factor (BDNF) in depressed patients with or without antidepressants”.. Biol Psychiatry.

[48] Okamoto T, Yoshimura R, Ikenouchi-Sugita A, Hori H, Umene-Nakano W, et al (2008). “Efficacy of electroconvulsive therapy is associated with changing blood levels of homovanillic acid and brain-derived neurotrophic factor (BDNF) in refractory depressed patients: a pilot study”.. Prog Neuropsychopharmacol Biol Psychiatry.

[49] Taylor SM (2008). “Electroconvulsive therapy, brain-derived neurotrophic factor, and possible neurorestorative benefit of the clinical application of electroconvulsive therapy”. The Journal of ECT..

[50] Mann JJ, Currier D (2006). Effects of genes and stress on the neurobiology of depression.. Int Rev Neurobiol.

[51] Krystal AD, Weiner RD (1999). EEG correlates of the response to ECT: a possible antidepressant role of brain-derived neurotrophic factor.. J ECT.

[52] Duman RS, Monteggia LM (2006). A neurotrophic model for stress-related mood disorders.. Biol Psychiatry.

[53] Aso E, Ozaita A, Valdizan EM, Ledent C, Pazos A (2008). BDNF impairment in the hippocampus is related to enhanced despair behavior in CB1 knockout mice.. J Neurochem.

[54] Jiang W, Zhang Y, Xiao L, Van Cleemput J, Ji SP (2005). Cannabinoids promote embryonic and adult hippocampus neurogenesis and produce anxiolytic- and antidepressant-like effects.. J Clin Invest.

[55] Domschke K, Lawford B, Laje G, Berger K, Young R (2010). Brain-derived neurotrophic factor (BDNF) gene: no major impact on antidepressant treatment response.. Int J Neuropsychopharmacol.

[56] Ren X, Dwivedi Y, Mondal AC, Pandey GN (2011). Cyclic-AMP response element binding protein (CREB) in the neutrophils of depressed patients.. Psychiatry Res.

[57] Reierson GW, Mastronardi CA, Licinio J, Wong ML (2009). Chronic imipramine downregulates cyclic AMP signaling in rat hippocampus.. Neuroreport.

[58] Monteleone P, Bifulco M, Maina G, Tortorella A, Gazzerro P (2010). Investigation of CNR1 and FAAH endocannabinoid gene polymorphisms in bipolar disorder and major depression.. Pharmacol Res.

[59] Domschke K, Dannlowski U, Ohrmann P, Lawford B, Bauer J (2008). Cannabinoid receptor 1 (CNR1) gene: impact on antidepressant treatment response and emotion processing in major depression.. Eur Neuropsychopharmacol.

